# Supplementary material for: Drought and Plant Community Composition Affect the Metabolic and Genotypic Diversity of Pseudomonas Strains in Grassland Soils
Source: Microorganisms. 2021 Aug 7;9(8):1677. doi: 10.3390/microorganisms9081677 (PMC8399733; doi:10.3390/microorganisms9081677)
Supplement: Supplementary file 1 [file microorganisms-09-01677-s001.zip › Supplementary_Figures.pdf]

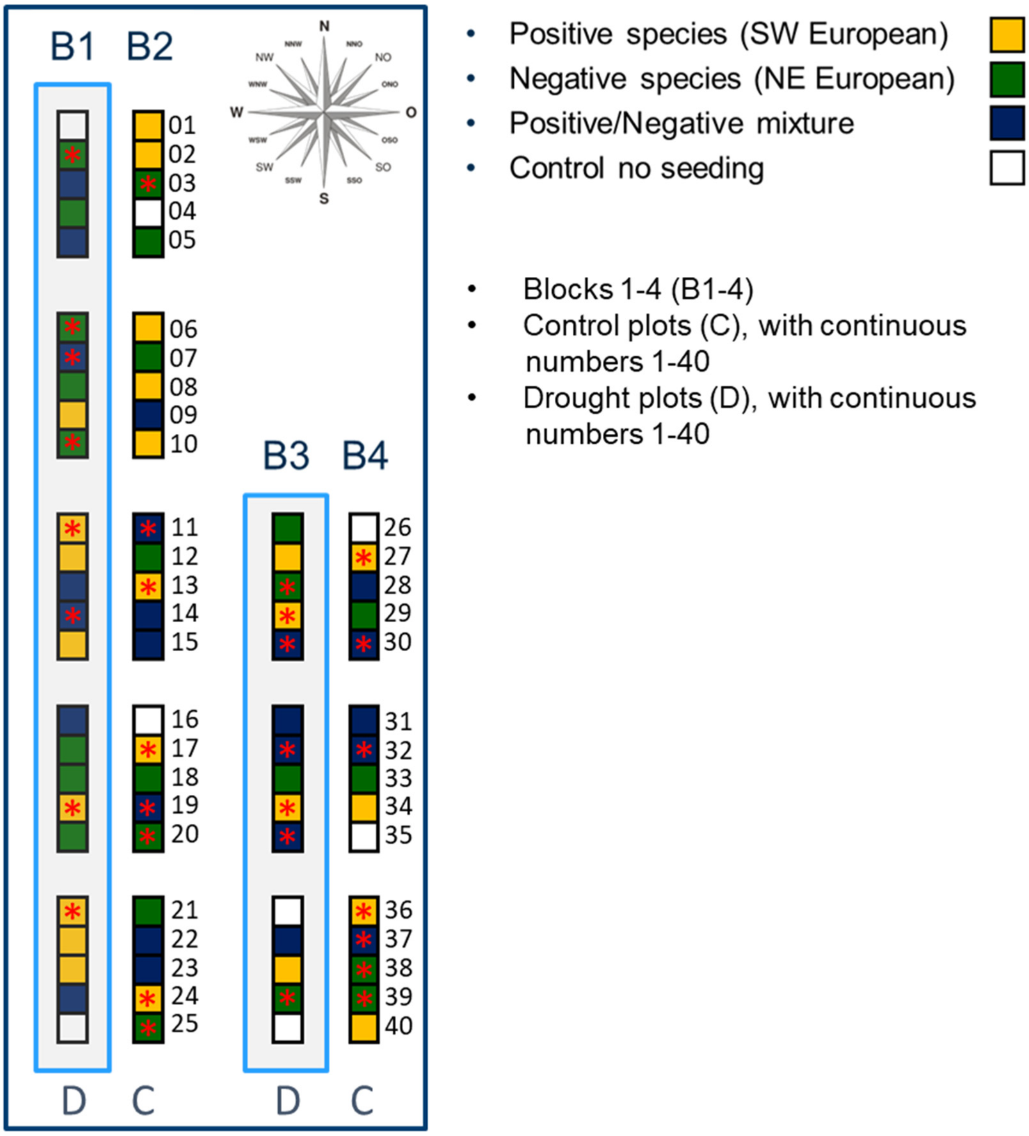

Figure S1 Experimental design of the Miniplot experiment. 80 small experimental plots (1 m²) arranged in 4 blocks. Block 1 and 3 were roofed over to simulate drought periods in May/June and August/September from 2010-2012. Block 2 and 4 were under ambient precipitation. To induce a plant treatment out of two species pools with each 32 species (8 grasses, 8 legumes, 8 small herbs and 8 tall herbs) 16 species were randomly taken. Special pools contained on the one hand species mainly distributed in NE Europe and species mainly distributed in SW Europe. Red asterisks mark the plots (n=30) used within the study.

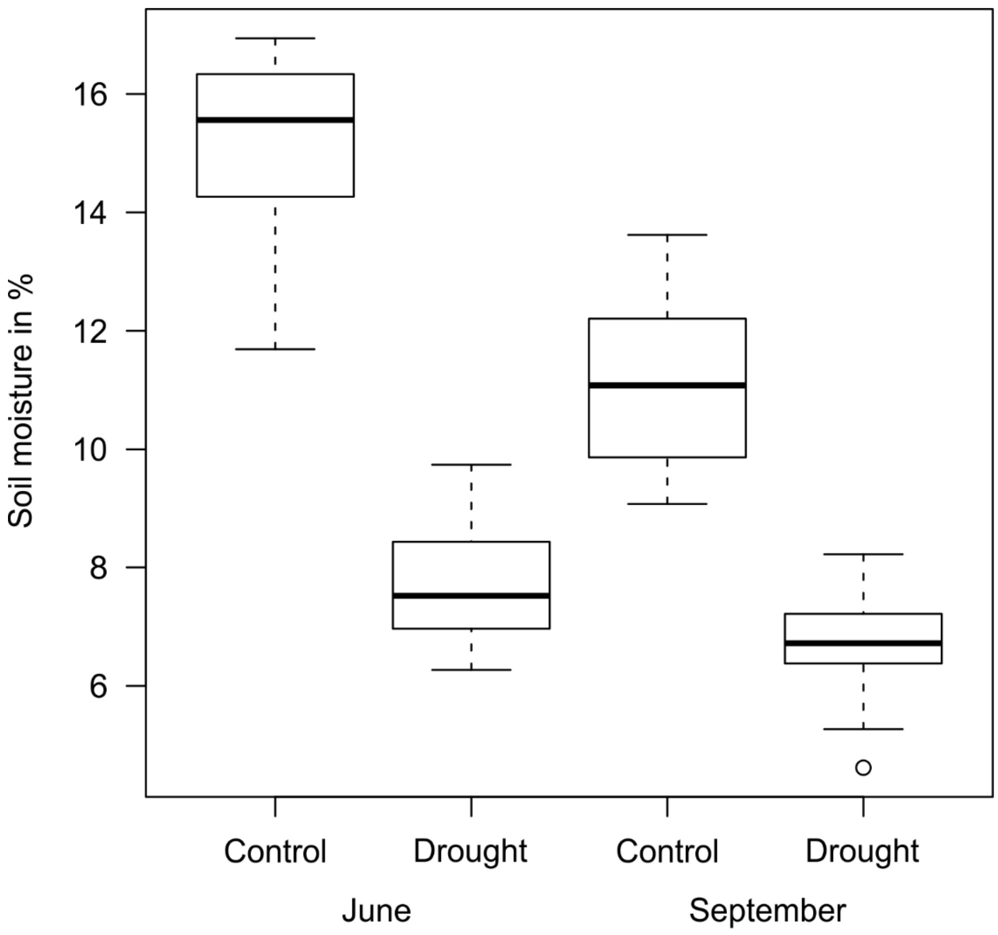

Figure S2 Gravimetric soil moisture under control and drought condition sampled in June and September.
